# Dissertatio Medica de Tussi Convulsiva Infantum

**Published:** 1781-12

**Authors:** 


IV. *Diſſertatio Medica de Tuſſi Convulſiva Infantum. Auctore* Carolo Strack, M. D. *Eminentiſſ. ac Celſiſ. Princ. Elect. Mogunt. Jud. Aul. Conſ. Inſtitut. Med. Profeſſ. publ. et ord. Facult. Med. Aſſeſſ. Electoral. Utilium Scient. Academ. Erford. et Princ. Haſſiac. Gieſſon. Socio.* 8vo. Moguntiæ, p. 28.

THE *tuſſis convulſiva*, or *hooping cough*, is one of thoſe diſeaſes, concerning which very different opinions have been maintained by medical authors. Some have ſuppoſed the principal ſeat of the complaint to be in the ſtomach; others in the lungs; while one of the lateſt writers on the ſubject alledges, that the cauſe of it is to be ſought for neither in the lungs nor the ſtomach, but in the inteſtinal canal. A pathology of the diſeaſe diſeaſe different from either of thoſe is given in the preſent performance, which is the production of a judicious and experienced writer.

Dr. Strack ſets out with defining the diſeaſe, after which he traces its ſymptoms and progreſs with great accuracy. It begins, he obſerves, with a ſlight cough, which neither quickens the pulſe, nor diſturbs the ſleep or the appetite of the patient. It is attended, however, with this peculiar circumſtance, that the throat is more affected by it than the thorax. In this manner it proceeds for a week, and ſometimes longer, when it becomes more violent and frequent, ſo as to diſturb the patient's breathing, and at the end of about the third week it ſeems to be at its height.

Of the diſeaſe at this period the author preſents us with the following deſcription, which we ſhall give in his own words:

" Ronchus in gutture auditur, qualem fere blandiens felis, ubi Heri circum crura mollis ſerpit, rotare conſueſcit. Sequitur titillatio ibidem, proximeque tuſſis vehementer erumpit, & ut incepit, ſpiritum convulſorio modo expellere atque ad extremum uſque halitum exprimere continuat: adeo ut eundem æger vix arripere intuſque reducere queat. Vere veluti illa tuſſis, quæ, quæ bibentem aliquem hominem, ſiquid potus illapſum in ejus glottidem eſt, præfocat atque convellit. Aut quemadmodum ira excandeſcentes pueri, qui cum plorare incipiunt, animam (ut matres loqui ſolent, & cuique notum eſt) retinent. Inde facies ſurſum pulſo atque in venas coacto ſanguine turget, profundeque rubra atque livida evenit. Ob quem utique colorem iſte tuſſi nomen lividæ *(der blaue Huſten)* a noſtratibus datum eſt. Et cum tali labore pulſus ſupra modum ex pulmone atque ad ſuffocationem uſque expreſſus omnis aër eſt, quærunt item pueri eundem magna vi laborioſoque conatu altum inter acutumque ſuſpirium arripere: idque nituntur levata cervice, fixiſque ſcapulis, atque magno oris hiatu, diductiſque trucum oculorum palpebris; aſthmaticorum inſtar. Ob id panſa brachia parieti opponere ſolent, ſcilicet ut tali fulcimento adjuti aërem fortius trahere queant; quem ut traxerunt, ſimiliter iterum per tuſſis violentiam ad extremum uſque ſpiritum extendunt; iterumque vi atque anxia agitatione revellunt. Sic queſexies deciesve repetitis continuo vicibus. Horribile profecto viſu & metu plenum ſpectaculum!

" Finito iſto tormento ægri laſſi ſunt, & membra fatigata habent, atque obdormiſcunt. Vere veluti veluti qui a morbo caduco torpent, atque somno opprimuntur."

The patient experiences, we are told, from twenty to thirty of these attacks every day. During the first weeks of the disease only a thin lymph is discharged from the throat, fauces, and mouth; but by degrees the mucus becomes of a thicker consistence, resembling the white of an egg, and is coughed up with great difficulty. After this follows a thick, white discharge, not unlike the cream of milk coagulated, and in proportion as this is ejected the cough becomes milder and less frequent, gradually decreasing for the space of two or three weeks. At length, observes our author, only a slight cough remains, which comes on once or twice every evening, continuing to return in this manner during two or three months if the disorder is left to itself.

With regard to the cause of this disease, Dr. Strack is persuaded, that it is seated in the glands of the fauces and glottis, particularly in those which are placed between the larynx and the basis of the tongue. That the seat of the complaint is not below the larynx or in the lungs themselves, he thinks probable, because in the inintervals of coughing the breathing is perfectly free and unaffected, and because the cough itself is exactly similar to that which is produced by irritating the larynx, as happens, for instance, when any of our food or drink passes into it. That the source of the disease is to be found in the stomach, he supposes to be equally unlikely, the vomiting which is brought on by the cough being the effect, not of any morbid state of the stomach, but merely of the cause just now mentioned, the irritation of the glottis. As a further proof of this, he adds, that the patients have no foulness of the tongue, no sense of oppression after meals, both which circumstances would take place were the stomach affected.

After having explained his ideas concerning the seat of the disease, our author proceeds to point out the two stages which he thinks are observable in its progress. These are the *stadium cruditatis*, and the *stadium concoctionis*, the latter he describes as taking place when the cough begins to abate, and when the mucus brought up is white and grumous. The more it exhibits of this clotted appearance the more complete is said to be the concoction.

During the first of these stages, the author contents himself, if the patient is an infant, with preprefcribing a linctus compofed of two drachms of *ol. amygdal.* mixed with yolk of egg, and an ounce of fyrup of manna with the addition of two grains of *fulphur aurat. antimon.* Of this medicine fmall dofes are directed to be administered frequently. The bowels are to be occafionally emptied by a glyfter; and when the fymptoms have been violent he has experienced good effects from a blifter between the fhoulders, and from venæfection.

The food during this period, he obferves, fhould be light, eafy of digeftion, and taken in fmall quantities, fo as to avoid diftending the ftomach. Whenever the patient coughs violently, and the mucus is heard loofened and rattling in the throat, he recommends the introducing a finger into the mouth in order to bring it away, and if between the fits of coughing there appears to be danger of fuffocation, we are advifed to blow ftrongly into the patient's mouth in order to diftend the lungs. For the fame reafon large, airy apartments are recommended, and in bed he advifes the patients to lie lightly covered, without curtains, and to fleep on one fide rather than in a fupine pofture.

When the difeafe has arrived at its fecond

 ftage,

ſtage, Dr. Strack preſcribes five grains of ipecacoanha as an emetic, after which he returns to the uſe of the linctus, and at the end of ſeven or eight days repeats the vomit. After this ſecond puke he has generally obſerved, that the cough and other ſymptoms have been almoſt entirely removed, and a mild purgative has been ſufficient to complete the cure. By this mode of treatment he aſſures us, that in general the diſeaſe has ſeldom extended itſelf beyond four weeks.

Towards the end of the work he takes occaſion to remark, that he has never ſeen any one attacked a ſecond time with this diſeaſe.

In an appendix to this little work the author preſents us with ſeveral aphoriſms under the title of *Poſitiones*, from which we ſhall ſelect the following:

"Qui choreæ ſancti Viti cauſam in nervis quærunt, longe a recto aberrant."

"Qui pueri cum magnis genitalibus & pendulis teſticulis naſcuntur, et denſa ſupercilia et claucos oculos habent, hi ante loquelam tabe conſumuntur."

"Viri toroſi, denſam et craſſam cutim habentes pilis ſetoſis hirtam, barbam item denſam, fortesque capillos, ſi ſimul latæ et humilis ſtaturæ

turæ ſunt, ut plurimum 60 annos non ſupervivunt. Moriuntur autem vel ob corruptum hepar, vel ob hydropem."

" Glabri et delicatiores, ſi ſobrie vivunt, ſeniores fiunt."

" Cicuta ſi vel urinam vel ſudorem cum prurìtu pellit, juvat. Si non, non."

